# Modelling of strategies for genetic control of scrapie in sheep: The importance of population structure

**DOI:** 10.1371/journal.pone.0195009

**Published:** 2018-03-27

**Authors:** Thomas J. Hagenaars, Marielle B. Melchior, Jack J. Windig, Alex Bossers, Aart Davidse, Fred G. van Zijderveld

**Affiliations:** 1 Wageningen Bioveterinary Research, Lelystad, The Netherlands; 2 Wageningen Livestock Research, Wageningen, The Netherlands; University of Lincoln, UNITED KINGDOM

## Abstract

Scrapie is a transmissible spongiform encephalopathy in sheep and an example of a disease that may be controlled through breeding for disease resistance. Member states of the European Union have introduced strategies for breeding against scrapie based on the selection of genetically resistant breeding rams. An ambitious strategy adopted in The Netherlands consisted of selecting resistant rams for breeding throughout both breeding and production sectors. Mathematical modelling of the effect of a breeding program on the spreading capacity of scrapie in a national flock is needed for making assessments on how long a breeding strategy needs to be maintained to achieve disease control. Here we describe such a model applied to the Dutch situation, with the use of data on the genetic content of the Dutch sheep population as well as on scrapie occurrence in this population. We show that the time needed for obtaining scrapie control depends crucially on two parameters measuring sheep population structure: the between-flock heterogeneity in genotype frequencies, and the heterogeneity of mixing (contact rates) between sheep flocks. Estimating the first parameter from Dutch genetic survey data and assuming scenario values for the second one, enables model prediction of the time needed to achieve scrapie control in The Netherlands.

## Introduction

Scrapie is a fatal infectious neurodegenerative disease for which susceptibility is associated with polymorphisms in the ovine prion protein (PrP) gene. Polymorphisms at codons 136 (A/V), 154 (R/H) and 171 (Q/R/H) largely determine resistance to scrapie with the VRQ allele being most susceptible, and the ARR allele being resistant to classical scrapie [1–3]. Based on selective breeding for resistance, national eradication programs have been implemented in several countries in Europe, including Great Britain [4–9] and The Netherlands [10]. The intensity of selective breeding varies between countries. One of the most ambitious programs was implemented in the Netherlands, where selection of rams with the ARR/ARR genotype for breeding started in 1998 (voluntary basis) and was obligatory for all sheep farmers from October 2004 to June 2007 [11].

The attempt to eliminate scrapie in this way in The Netherlands can be seen as an instructive attempt to control a widespread infectious disease by breeding, rather than by the more usual vaccination and stamping-out strategies. Clearly, the level of compliance to the selection of scrapie resistant rams for breeding is an important determinant of the effectiveness of the program. However, full compliance may not be needed if a lower than 100% level of resistance is sufficient for control. From an epidemiological viewpoint national scrapie control can be considered achieved when the population-level basic reproduction number *R*_0_ has been reduced to below unity, the threshold value for epidemic spread. The frequency of the ARR allele for which this threshold value is attained is the minimum ARR allele frequency for scrapie control, a concept analogous to that of a critical vaccination coverage [12]. A previous analysis [11] has provided evidence that the Dutch scrapie control program up to now has produced both an increase in the prevalence of scrapie resistant genotypes and a reduction in scrapie transmission.

Our first objective is to predict the minimum frequency of the resistant allele in the sheep population needed to achieve scrapie control. The second objective is to calculate, under different compliance scenarios, how long the ram selection program needs to be maintained to reach the minimum ARR allele frequency and achieve scrapie control. These two objectives are achieved by constructing a combined genetic and epidemiological model, and by using this model to predict the time development of genotype frequencies and reproduction number *R*_0_ at a national scale. Our model calculation of *R*_0_ for scrapie requires quantification of two important between-flock heterogeneities. The first one is the variation in within-flock genotype frequencies. The level of such variation is important for the prospects for scrapie control: the higher the abundance of farms with, initially, relatively low frequencies of resistant animals, the longer the breeding program may need to be sustained to reduce *R*_0_ to below unity (and, correspondingly, the higher the population-level minimum ARR allele frequency will be). In Ref. [10] a genotyping survey of farms in the production sector, that accounts for over 90% of the Dutch sheep population, was carried out to gain information on the variation between farms with respect to genotype composition. We will use a within-flock transmission model developed in Ref. [13] to translate the distribution of within-flock genotype frequencies into a distribution of within-flock *R*_0_ values which in turn serves as a basis for the modelling of between-flock transmission. The second important between-flock heterogeneity is of contact rates (mixing) between sheep flocks. We will use a simple model characterizing this heterogeneity with a single parameter. Due to a lack of data, the value of this parameter is unknown; our model predictions will therefore be based on assuming scenario values.

Recently further quantitative trait loci influencing resistance to scrapie have been identified [2] as well as PrP gene polymorphisms at codons other than 136, 154 and 171 having a protective effect [3]. These findings widen the range of options for the design of breeding programmes, which could be of relevance in particular in breeds with a low frequency of the ARR allele. We do not model these polymorphisms here as they were not utilised in the Dutch breeding programme.

## Material and methods

### Surveillance data

We use surveillance data consisting of the scrapie test results accumulated within the Dutch active surveillance on TSEs in sheep (from 2002 onwards), and of a yearly random genotyping sample from this active surveillance (from 2005 onwards), both from the healthy-slaughter and the fallen-stock samples. Details on the sampling strategy, genotyping technique and rapid test used are given in Ref. [11]. The test sensitivity in detecting scrapie infection in animals without ARR allele is unknown. Evaluated on scrapie cases confirmed by Western Blot of the brainstem the test sensitivity is close to 95% [14]. However, test sensitivity in the surveillance is expected to be lower as early on in the incubation period scrapie infection has not yet propagated to the brainstem [15]. As the rate of propagation to the brainstem is also genotype dependent, test sensitivity may therefore also be expected to depend on genotype. Detected scrapie prevalence in Dutch culled flocks gives an indication of the minimum value of the sensitivity [13].

### Culled-flocks data

The culled-flocks data (2003–2008) consist of scrapie genotyping results and scrapie infection test results in animals that were culled, as part of the mandatory scrapie control efforts, on flocks of origin of scrapie index cases. For details on genotyping and testing see [11]. Immunohistochemistry (IHC) was used for confirmation of the positive cases detected using the rapid test. IHC and Western blotting were used to discriminate between classical and atypical scrapie.

### Genotyping survey data

In 2007 a postal and genotyping survey in Dutch sheep flocks was carried out. The results were described in Ref. [10]. From 689 farms that completed the postal survey, 168 accepted the offer to genotype (part of) their animals. A maximum of 35 ewes were blood sampled per farm, and samples were taken proportionally per birth year cohort. If farmers owned less than 35 ewes, a maximum of 5 rams could be sampled too. Samples were sent to the Central Veterinary Institute in Lelystad (Now Wageningen Bioveterinary Research, Lelystad) for analysis of the polymorphisms at the PrP gene codons 136, 154 and 171 through Taqman probe analysis. A total of 3314 sheep were genotyped, including 3207 ewes born between 1995 and 2007. For further details on this survey we refer to Ref. [10].

### Modelling: Broad strategy

We model the Dutch national sheep population as a population of sheep flocks that vary in genetic content, distinguishing two levels of transmission: within a flock and between flocks. For a review of previous within-flock and between-flock scrapie transmission modelling see [16]. The importance of taking into account the genetic variation between flocks can be illustrated as follows. Let us assume for definiteness that large within-flock scrapie outbreaks would be precluded if the ARR allele frequency in the flock exceeds 80%, and that the overall ARR allele frequency in the population is 85%. Then, if half of the flocks would have an ARR frequency of 100% and the other half one of 70%, large outbreaks were still possible in 50% of flocks, in contrast to a situation without variation, in which all flocks would have the same allele frequency of 85% and in which large outbreaks were not possible in any flock. We note that apart from the between-flock differences in genetics, another reason why it is natural to distinguish the two levels of transmission in the population is that contacts between sheep residing within one and the same flock are more intensive than between animals residing in different flocks.

The within-flock model calculates the within-flock reproduction number, denoted in this paper by $R_{0}^{w}$, from the genotype distribution in the flock. This is based on a model developed in Ref. [13], which is parameterized using genotyping and case data from Dutch flocks culled under EU statutory control measures [13]. An initial frequency distribution of within-flock $R_{0}^{w}$ values is based on both a farm genotyping survey [10] and a genotyping sample from the active surveillance [11]. Starting from this initial distribution, we use the within-flock transmission model to calculate how the distribution evolves in time for a given level of compliance to the ram selection program. From this, the between-flock model in turn calculates the time evolution of the between-flock *R*_0_. The latter parameter represents the population-level *R*_0_, and when it drops below unity, national scrapie control has (by definition) been achieved. The ARR allele frequency for which *R*_0_ = 1 is the minimum frequency required for scrapie control. The starting value of the population-level *R*_0_, characterizing the situation in 2008, is based on scrapie incidence data in the Dutch active surveillance. When the between-flock *R*_0_ ≤ 1, isolated within-flock outbreaks of scrapie may still occur with varying duration [17] but no major between-flock spread will be possible. For a field study showing the success of selective breeding to control scrapie *at the flock level* see [18].

### Modelling within-flock transmission

Our purpose is to model the flock-level scrapie transmission potential, as quantified by the within-flock basic reproduction number, in dependence of the within-flock genotype frequencies. We use an SI-type within-flock transmission model with homogeneous mixing between sheep of different genotype, in which we assume that genotypes differ both in susceptibility and in infectiousness; this model was developed in Ref. [13]. We denote by *f*_*γ*_ the proportion of animals in the flock that has genotype *γ*, by *g*_*γ*_ the relative susceptibility of genotype *γ*, and by *h*_*γ*_ the relative infectiousness of genotype *γ*. Finally, we denote the absolute scale of transmission by a dimensionless parameter *β*. Then the definition of the reproduction number [19] leads to the following expression within-flock $R_{0}^{w}$:

$$\begin{array}{l} R_{0}^{w}=\beta Q_{0}^{w},\\ Q_{0}^{w}=\sum_{\gamma }f_{\gamma }g_{\gamma }h_{\gamma }. \end{array}$$

This expression defines $R_{0}^{w}$ as a weighted average of the product *βg*_*γ*_*h*_*γ*_, with the genotype frequencies *f*_*γ*_ as weighting factors. The values of the parameter products *g*_*γ*_*h*_*γ*_ used are based on setting the *g*_*γ*_ equal to the relative scrapie risks in different genotypes as estimated from culled-flocks data in Ref. [11] (the values of *g*_γ_ used are given in Table 1 of Ref. [13]) and on setting the parameters *h*_*γ*_ equal to one for all genotypes except for those with at least one ARR allele, for which *h*_*γ*_ is set to zero. This approximation is based on the assumption motivated in [13] that the contribution of ARR/VRQ and ARR/ARQ animals to $R_{0}^{w}$ is negligible. The parameter *β* incorporates the variation in $R_{0}^{w}$ due to causes different from the genetic content of the flock, such as lambing practice; it may therefore differ between flocks. The variation in *β* is described using a Weibull distribution, the two parameters of which were estimated in Ref. [13].

We calculate the effect on $R_{0}^{w}$ of a breeding program based on ram selection as follows. When a flock is subject to ARR/ARR ram selection, the newborn lambs, from which replacement stock will be selected, have at least one ARR allele. It follows that, if we neglect bought-in replacement stock (as these are typically small in number [10]), replacement animals will contribute negligibly to $R_{0}^{w}$. Thus, assuming that all age categories are subject to the same replacement rate, the expected change in $R_{0}^{w}$ between year *t* and year *t*+1 due to ram selection equals:

$$R_{0}^{w}\left(t+1\right)-R_{0}^{w}\left(t\right)=-rR_{0}^{w}\left(t\right),$$ (1)

where *r* denotes the yearly replacement rate. The expected change in the frequency *f*_*ARR*_ of the ARR allele in the flock can also be expressed in terms of *r*, as follows:

$$f_{\mathrm{ARR}}\left(t+1\right)-f_{\mathrm{ARR}}\left(t\right)=\frac{r}{2}\left(1-f_{ARR}\left(t\right)\right).$$ (2)

This relationship can be derived by noting that *f*_*ARR*_ changes due to replacement animals having a different ARR allele frequency than the ewes they are born to; as ARR/ARR rams are selected for breeding, the frequency of non-ARR alleles in the newborns is one half of that in the ewes.

### Modelling between-flock transmission

In the between-flock transmission model we consider a population of flocks for which the $R_{0}^{w}$ is drawn from a distribution *PDF*_*t*_(*R*_0_^*w*^). In order to derive this distribution for the year *t* = 2008, the starting point for our predictive calculations, we calculate $Q_{0}^{w}=R_{0}^{w}/\beta$ for each of the 168 flocks of the genotyping survey, and determine a distribution model that provides a good match to the histogram of 168 values. Subsequently, we obtain *PDF*_2008_(*R*_0_^*w*^) from this distribution and from the Weibull model distribution for *β* [13] as the distribution of the product of $Q_{0}^{w}$ and *β*.

We consider flocks with $R_{0}^{w}$ above one to be susceptible to flock-to-flock transmission, and flocks with $R_{0}^{w}$ below one to be resistant. I.e., we define *s*(*t*), the proportion of susceptible farms at time *t* as:

$$s\left(t\right)=\int_{1}^{\infty }PDF_{t}\left(R_{0}^{w}\right)dR_{0}^{w}.$$ (3)

We note that for the time evolution of *s*(*t*) it does not matter to which extent the farms with $R_{0}^{w}$ already below one comply with the breeding program; only the compliance of farms with $R_{0}^{w}$ above one matters. We also note that once a breeding program has run for some time, as is the case in The Netherlands at t = 2008, the average compliance of the farms with $R_{0}^{w}$ above one will be less than that of farms with $R_{0}^{w}$ below one, as the breeding program makes the average $R_{0}^{w}$ of the compliant farms go down. We take this effect into account by a model, detailed in the SI, that calculates, from an overall compliance (the “compliance” for which we list the scenario values in the Result section), the compliance of farms which have an $R_{0}^{w}$ above one in 2008.

For the compliant part of the population, the relationship expressed by Eq (1) implies that one year of ram selection reduces each $R_{0}^{w}$ with a factor 1 −*r*. Therefore, starting from the $R_{0}^{w}$ distribution for a given year *t*, one year of ram selection with a compliance *c* produces the following new distribution:

$$PDF_{t+1}\left(R_{0}{}^{w}\right)=\left(1-c\right)PDF_{t}\left(R_{0}^{w}\right)+\frac{c}{1-r}PDF_{t}\left(R_{0}^{w}/\left(1-r\right)\right).$$

With the compliance *c* we denote the proportion of farms, within the farms that have an $R_{0}^{w}$ above one in 2008, that comply with the ram selection program. In this model the $R_{0}^{w}$ distribution for non-compliant farms is assumed to be stationary. This is a conservative approximation, as there will be some dissemination of resistant alleles into non-compliant farms when they buy-in replacement ewes from compliant flocks. As the postal survey in 2007 [10] indicated that only 20% of Dutch sheep farms frequently purchase ewes, we expect this dissemination effect to be relatively small. Our model also neglects the effect that the culling of detected scrapie flocks, through removing susceptible alleles, will have on the $R_{0}^{w}$ distribution. This effect is expected to be small due to the low yearly detection probability of affected flocks based on the arguments given in the additional file of Ref. [11].

In order to approximately account for heterogeneities of mixing between flocks that exist as a result of e.g. regionality of contacts and between-farm differences in trading of animals, breeds present on the farm, and levels of shared grazing, we introduce a single mixing parameter *α*. In absence of data on the mixing between Dutch flocks, more detailed modelling of the heterogeneities would introduce more parameters with unknown values. The parameter *α* enters in the relationship between the population-level *R*_0_ and the observed prevalence of infected farms, that we assume to be as follows:

$$R_{0}=\alpha \left(\frac{1}{1-i^{*}}-1\right)+1.$$ (4)

Here i* is the (endemic) prevalence of infected farms within the subpopulation of farms with $R_{0}^{w}>1$, and *α≥*1. For the case *α* = 1 (homogeneous mixing) the model reduces to a well-known result for the SIR model in endemic equilibrium. For *α>*1 it provides a simple, phenomenological expression for heterogeneous mixing, as heterogeneous mixing causes *R*_0_ to be higher than the value often calculated using the well-known result for homogeneous mixing [20]. In an SIR model of a population of size *N* in which a proportion *f* of individuals are immunized at birth or are genetically immune against the infection, the relationship between the population-level *R*_0_ and the observed endemic prevalence of infection is given by $R_{0}=\frac{1}{1-\alpha i^{*}}$, with *α* = 1/(1 − *f*). For sufficiently small αi* this relationship coincides with Eq (4) to a good approximation. In terms of heterogeneous mixing, the model given by Eq (4) can therefore be thought of as approximately describing a population in which a core group of size *N*/*α* is responsible for the bulk of the transmission due to high contact rates, and the remaining group of size *N* − *N*/*α* hardly contributes due to low contact rates. For example, a value of *α* = 4 can be roughly interpreted as a situation in which 25 percent of the non-resistant flocks are responsible for the bulk of between-flock transmission.

The extrapolation of the reproduction number in time starting from its estimated value for 2008 (denoted as *R*_0_(2008)) is carried out as follows:

$$R_{0}\left(t\right)=\frac{s\left(t\right)}{s\left(2008\right)}R_{0}\left(2008\right).$$ (5)

Here it is assumed that when *α*>1, the compliance as well as the $R_{0}^{w}$ distribution are the same across flocks of different mixing types. The ARR allele frequency for which *R*_0_(*t*) = 1 is the minimum frequency required for scrapie control.

The value *R*_0_(2008) is calculated by applying Eq (5) to an endemic situation observed in the surveillance in 2002–2005, estimating the prevalence i* for *t* = 2005 from the prevalence in the active surveillance and in culled flocks and extrapolating *R*_0_(2005) to *R*_0_(2008) as detailed in the Supplementary Information.

**Fig 1:**
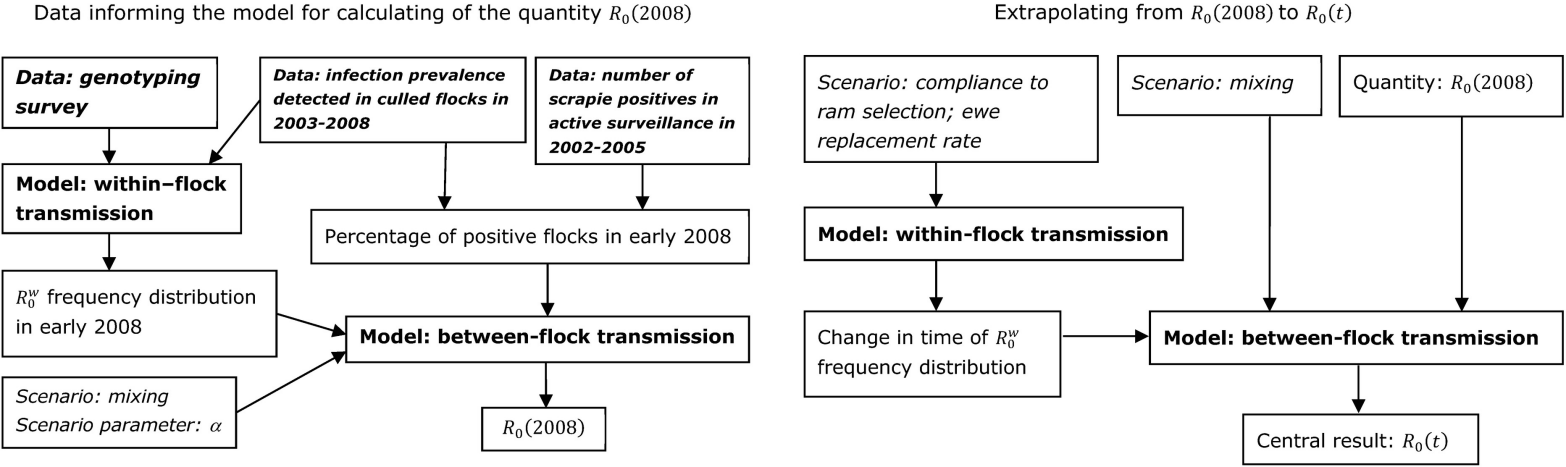
Calculation approach. Flow diagrams summarizing the calculation approach.

A flow diagram summarizing our calculation approach, including the within- and between-flock modelling parts as well as the different data sources used, is given in Fig 1.

## Results

**Fig 2:**
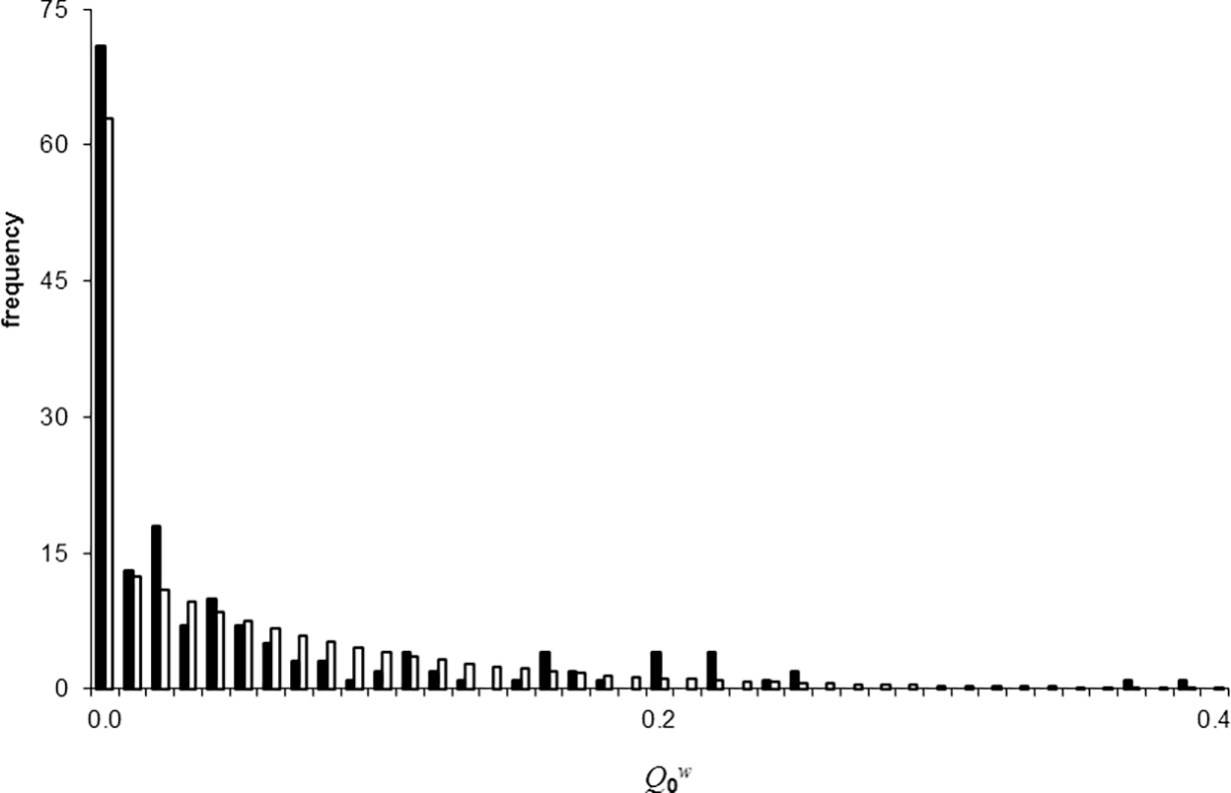
Distribution of weighted flock genotype frequencies. Histogram of the quantity $Q_{0}^{w}=R_{0}^{w}/\beta$. Black bars: as calculated using the within-flock model of Ref. [13] from the genotyping survey results in 168 flocks. White bars: model distribution (for details see S2 File).

In Fig 2 we present a histogram of the quantity $Q_{0}^{w}=R_{0}^{w}/\beta$ as calculated with the within-flock model from the genotyping survey data in 168 flocks (black bars). The results show that there is much variation in $Q_{0}^{w}$ between flocks, in line with the variation in genotype frequencies discussed in [10]. The white bars in Fig 2 represent a model distribution fitted to the data histogram. The distribution is of exponential form with an additional probability at $Q_{0}^{w}=0$ (for details see SI).

**Fig 3:**
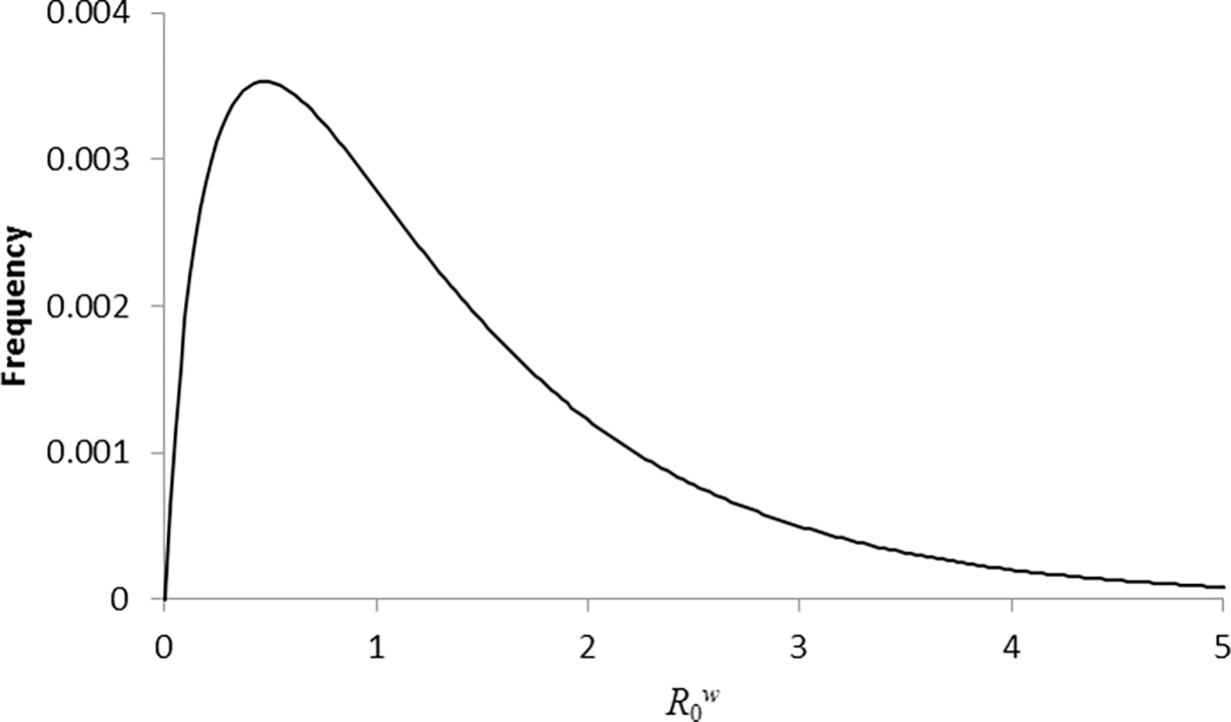
Distribution of within-flock reproduction number. Model distribution for $R_{0}^{w}$ obtained from the model distribution for $Q_{0}^{w}$ shown in Fig 2 and the Weibull model distribution for *β* estimated in Ref. [13].

As described in the Methods section, the model distribution for $Q_{0}^{w}$ shown in Fig 2 and the Weibull model distribution for *β* estimated in Ref. [13] together determine the model distribution for $R_{0}^{w}$. The part of this distribution relating non-zero $R_{0}^{w}$ values is shown in Fig 3. According to the model, 37.3% of Dutch flocks had an $R_{0}^{w}$ above one in early 2008, i.e. 37.3% of flocks were susceptible to epidemic within-flock scrapie spread. The tail of the $R_{0}^{w}$ distribution in Fig 3 corresponds to flocks that, as they have highest scrapie transmission potential, would require the longest period of selective breeding to bring $R_{0}^{w}$ below one. This tail is therefore an important determinant of the prospects for obtaining population-level scrapie control.

**Fig 4:**
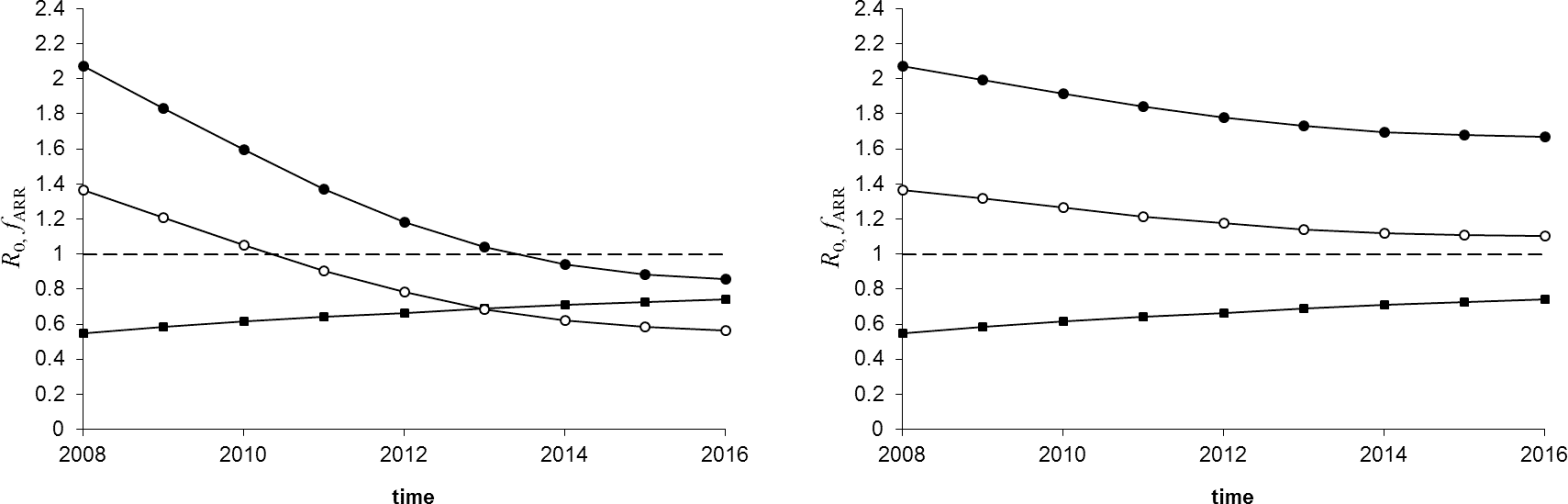
Predicted effect of breeding programme. Predicted *R*_0_ between Dutch flocks (circles) and ARR allele frequency *f*_*ARR*_ (squares) as a function of time; assumed is a yearly replacement rate of 20% (r = 20%). The line with open (closed) circles corresponds to *α* = 2 (*α* = 4).The dashed line indicates the critical value *R*_0_ = 1. Left panel: Compliance to ram selection of 75%; Right panel: Compliance to ram selection of 35%.

Model extrapolation results for *R*_0_(*t*) under the two scenarios of compliance c = 75% (“high compliance”) and c = 35% (low compliance) are shown in Fig 4. In both scenarios we assume that r = 0.2, which is a plausible estimate for the mean replacement rate in Dutch sheep farming [21].

In order to explore the sensitivity of outcome to the uncertainty in *α* we use two alternative moderately heterogeneous mixing scenarios, defined by *α* = 2 and *α* = 4. We also show the time evolution of the overall ARR allele frequency in the Dutch sheep population, obtained by applying Eq (2) to the compliant part of the population. The results for *α* = 2 suggest that for the high-compliance scenario, scrapie control was achieved in The Netherlands by 2011, when the overall ARR frequency exceeds a minimum of approximately 63% (Fig 4A). In contrast, for a value of *α* = 4 results in a minimum overall ARR frequency of approximately 70%, obtained by 2014. This sensitivity analysis thus shows that both the minimum ARR frequency and the time by when control is achieved are sensitive to the uncertainty in *α*. The minimum frequency is also (very) sensitive to the compliance level. For a compliance of only 35% (Fig 4B) our model suggests that scrapie control is never reached, as this compliance level is insufficient for reaching *R*_0_ ≤ 1.

**Fig 5:**
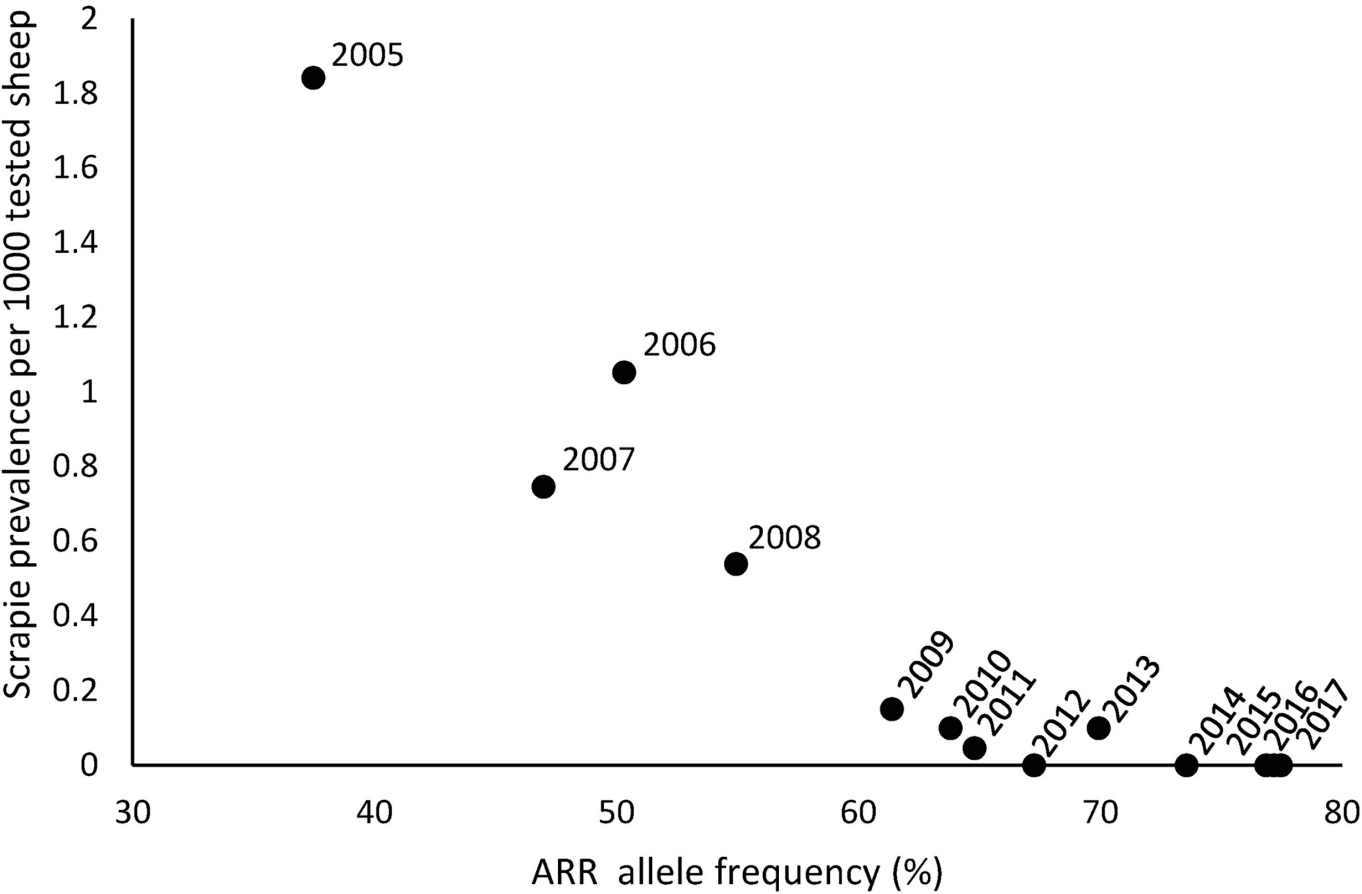
Active surveillance results for the Netherlands. Scrapie prevalence in the active surveillance in the years 2005 to 2017 against ARR allele frequency as measured in a yearly random sample from the surveillance.

Our results suggest that with a compliance of 75% scrapie control would have been obtained approximately between 2010 and 2014 for moderately heterogeneous mixing. This prediction is consistent with recent downward trends in scrapie incidence observed in the Dutch active surveillance [22] shown in Fig 5. The increase in *f*_*ARR*_ observed in Fig 5 in the period 2008–2013 is consistent with a compliance to selective breeding of 75%, and the low prevalence of scrapie in tested animals in recent years is a suggestive indication that scrapie control may have been achieved.

## Discussion

We have developed a model describing within-flock and between-flock scrapie transmission as well as the effect on transmission of changes in genotype frequencies due to selection of ARR/ARR rams for breeding. Using this model, we have calculated the minimum ARR allele frequency to obtain classical scrapie control in The Netherlands. The results suggest that for (overall) compliance of 75%, scrapie control is achieved in The Netherlands when the overall ARR frequency exceeds a minimum value in the range of 63 to 70 percent across scenarios assuming moderate heterogeneity of between-flock mixing. These predictions are consistent with more recent surveillance data that suggest that the current (2017) resistant allele frequency is approximately 76% percent and that current scrapie prevalence is very low.

By a sensitivity analysis we have shown that the model prediction for the time needed for obtaining scrapie control is dependent in particular on the heterogeneity of between-flock mixing, which usually is difficult to estimate due to a paucity of data. The stronger this heterogeneity, the slower the decline of the population-level basic reproduction number, and therefore the slower the progress towards scrapie control through selective breeding. For the Netherlands, the consistency of recent surveillance data with the model scenarios assuming moderate heterogeneity of between-flock mixing suggests that the mixing of Dutch population of sheep flocks is characterized by a weak or moderate level of heterogeneity. Another important determinant is the between-flock heterogeneity in genotype frequencies, which we have quantified using random genotyping survey data. Finally, the level of compliance to ram selection is an important determinant of the predicted minimum ARR allele frequency to obtain classical scrapie control; in particular, if the compliance is too low, scrapie control will never be reached. The approximate overall compliance to ram selection in The Netherlands is 75% as can be deduced from the observed increase in the ARR allele frequency found in random genotyping samples from the active surveillance. In our model calculation we have not addressed what would be the effect on the minimum ARR allele frequency required for scrapie control in the Dutch sheep population if there would be changes to the surveillance intensity and/or the statutory scrapie flock culling policy. As has been argued in Ref. [11], these latter measures are thought to have only a minor influence (of a few percent) on the scrapie transmission risks as measured by the population-level *R*_0_. Therefore, although in principle this minimum ARR frequency would rise when the number of animals tested is reduced (as has been the case in The Netherlands since January 2014) and/or when the statutory control measures were ceased in future, such a rise would be expected to be at most a few percent.

The attempt to eliminate scrapie by selective breeding in The Netherlands can be seen as an instructive attempt to control a widespread infectious disease by breeding, rather than by the more usual vaccination and stamping-out strategies. We hope that our modelling approach and results are also instructive to readers interested in other host-pathogen systems in which genetic changes in the population impact on pathogen spread.

## Supporting information

S1 File Genetic survey data. By-flock genotyping results for a sample from 168 random Dutch sheep farms in 2007 [10].
(XLSX)

S2 File Descriptions of modelling details. Details of the fitting of a distribution to the genetic survey data; the estimation of i* (2005); and the extrapolation from *R*_0_(2005) to *R*_0_(2008).
(DOCX)

S1 Table Active scrapie surveillance and genotyping in The Netherlands. Numbers of animals tested in healthy slaughter and fallen stock streams by year; size of the yearly random samples taken for genotyping; number of scrapie cases by year in active surveillance and ARR frequency in the genotyped sample by year.
(DOCX)
