# Supplementary material for: Modelling of strategies for genetic control of scrapie in sheep: The importance of population structure
Source: PLoS One. 2018 Mar 27;13(3):e0195009. doi: 10.1371/journal.pone.0195009 (PMC5871008; doi:10.1371/journal.pone.0195009)
Supplement: S2 File — Details of the fitting of a distribution to the genetic survey data; the estimation of i* (2005); and the extrapolation from R0(2005) to R0(2008). (DOCX) [file pone.0195009.s002.docx]

**S2 File.** Descriptions of modelling details.

Supporting Information to ‘Modelling strategies for genetic control of scrapie in sheep: the importance of population structure’ by Hagenaars et al.

*Distribution fitted to genetic survey data*

This distribution is of the following form:

$PDF_{t=2008}\left(Q_{0}^{w}\right)=\frac{\lambda}{\kappa+1}\left(\frac{\kappa}{\lambda}\delta\left(Q_{0}^{w}\right)+\exp\left(-\lambda Q_{0}^{w}\right)\right)$. (S1)

Here *κ* and *λ* are fitting parameters, and *δ* is the Dirac delta distribution. The distribution displayed by the white bars in Figure 2 is the fitted model (S1), with fitted parameter values* κ*=0.41 and *λ*=12.6. These parameters are computed by requiring the following two conditions: to reproduce exactly (1) the proportion of flocks with $Q_{0}^{w}=0$in the genetic survey data, and (2) the tail of the genetic survey distribution defined by the proportion of flocks in the survey with $Q_{0}^{w}>1/\left\langle\beta\right\rangle$, with $\left\langle\beta\right\rangle$ the mean value of $\beta$in the within-flock model of Ref. [[10](#_ENREF_10)]. We note that the theoretical maximum value for $Q_{0}^{w}$ is equal to 1.31 (corresponding to the unrealistic case of a flock consisting of 100% VRQ homozygotes); however, our model probability density has dropped to negligibly small values already by $Q_{0}^{w}=0.6.$

Subsequently, we obtain $PDF_{2008}\left(R_{0}^{w}\right)$ from this distribution and from the Weibull model distribution for $\beta$ [[10](#_ENREF_10)] as the distribution of the product of $Q_{0}^{w}$and $\beta$.

*Estimation of* $i^{*}\left(2005\right)$

We note that surveillance results suggest an endemic situation in 2002-2005, with prevalence of approximately 2 per 1000 ewes tested [[8](#_ENREF_8)]. Our estimation of $i^{*}\left(2005\right)$, the prevalence of infected farms in year $2005$ from the infection incidence in the active surveillance is based on the following model:

$i^{*}\left(2005\right)=\frac{2P\left(\text{animal found positive in }2005\right)}{P\left(\text{animal originating from infected farm found positive}\right)}\frac{1}{s\left(2005\right)}$,

where $P\left(\text{animal found positive in year }2005\right)$ is a probability determined from the active surveillance and $P\left(\text{animal originating from infected farm found positive}\right)$ is a probability estimated from the culled-flocks data; the latter probability is estimated as 2.33 percent based on the data in Tables 6 and 8 in Ref. [[10](#_ENREF_10)]. The model is based on the observation that the probability of an animal being tested positive in the surveillance equals the probability that the farm it is originating from is infected times the probability that an animal tested on an infected farm becomes positive. However, as there are large between-flock differences in the scrapie prevalence, and higher prevalence enhances detection probability, the apparent mean prevalence in culled flocks will be biased upward. We correct for this by the factor 2 included in the numerator. It arises due to an exponential model that we assume for the distribution of within-flock prevalence. We note that if we assume that the unknown sensitivity of the rapid test (discussed in the main text) is the same for index cases as for further cases in affected farms, our result for the prevalence of infected farms is independent of that sensitivity.

*Model for calculating from an overall compliance the compliance of farms which have an $R_{0}^{w}$ above one in 2008*

We note that the compliance of farms which have an $R_{0}^{w}$ above one is defined by $c=\frac{c_{0}s_{c}}{c_{0}s_{c}+\left(1-c_{0}\right)s_{nc}}$,

with $c_{0}$ the overall compliance and$s_{c}\left(s_{nc}\right)$ the proportion of farms with $R_{0}^{w}>1$ within the compliant (non-compliant) sub-population of flocks, respectively. One year after the start of ram selection we may thus write:

$c\left(1\right)=\frac{c_{0}s_{c}\left(1\right)}{c_{0}s_{c}\left(1\right)+\left(1-c_{0}\right)s_{nc}}=\rho_{c}c$, (S2)

with $\rho_{c}=\frac{c\left(1\right)}{c}$ the factor with which compliance is reduced by one year of selective breeding, and $s_{c}\left(1\right)$ the proportion of farms with $R_{0}^{w}>1$ within the compliant sub-population of flocks one year after the start of selective breeding. Using Eq. (3) from the main text and noting that for the compliant part of the population, the relationship expressed by Eq. (1) implies that one year of ram selection reduces each $R_{0}^{w}$ with a factor $1-r$, we find that:

$s_{c}\left(1\right)=\frac{1}{\left(1-r\right)}\int_{1}^{\infty} PDF_{0}\left(\frac{R_{0}^{w}}{\left(1-r\right)}\right)dR_{0}^{w}$. (S3)

Here, $PDF_{0}\left(R_{0}^{w}\right)$ is the distribution of the within-flock reproduction number $R_{0}^{w}$ before the start of selective breeding. As the distribution of $R_{0}^{w}$in the non-compliant does not change, we have

$s_{nc}=\int_{1}^{\infty} PDF_{0}\left(R_{0}^{w}\right)dR_{0}^{w}=s\left(0\right)$. (S4)

To calculate the compliance $c$ for 2008, which is four years after the start of compulsory ram selection in The Netherlands, and onwards from a given overall compliance $c_{0}$ (assumed constant in time) we use the model:

$c\left(2008\right)=\left(\rho_{c}\right)^{4}c$, (S5)

in which we calculate $\rho_{c}$using Eq. (S2-S4) and substituting the model distribution $PDF_{2008}\left(R_{0}^{w}\right)$ shown in Figure 2 as an approximation for (the shape of) $PDF_{0}\left(R_{0}^{w}\right).$

*Extrapolating $R_{0}\left(2005\right)$ to* $R_{0}\left(2008\right)$

We relate $R_{0}\left(2008\right)$ to the estimated value for $R_{0}\left(2005\right)$ as follows:

$R_{0}\left(2008\right)=\frac{s\left(2008\right)}{s\left(2005\right)}R_{0}\left(2005\right)$,

in analogy with Eq. (5) in the main text. Based on following similar lines of reasoning as in the above paragraph, the model used for calculating the ratio $\frac{s\left(2008\right)}{s\left(2005\right)}$ is given by:

$$\frac{s\left(2008\right)}{s\left(2005\right)}=c\left(1\right)\left(\frac{s_{c}\left(1\right)}{s\left(0\right)}\right)^{3}+\left(1-c\left(1\right)\right).$$

Here $c\left(1\right)$ is calculated using an overall compliance of $c_{0}=0.75$ consistent with the random genotyping samples from the Dutch active surveillance.
