# Supplementary material for: Modelling of strategies for genetic control of scrapie in sheep: The importance of population structure
Source: PLoS One. 2018 Mar 27;13(3):e0195009. doi: 10.1371/journal.pone.0195009 (PMC5871008; doi:10.1371/journal.pone.0195009)
Supplement: S1 Table — Numbers of animals tested in healthy slaughter and fallen stock streams by year; size of the yearly random samples taken for genotyping; number of scrapie cases by year in active surveillance and ARR frequency in the genotyped sample by year. (DOCX) [file pone.0195009.s003.docx]

| **S1 Table. Active scrapie surveillance and genotyping in The Netherlands.** | | | | | | |  |  |  |  |  |  |  |  |  |  |
| --- | --- | --- | --- | --- | --- | --- | --- | --- | --- | --- | --- | --- | --- | --- | --- | --- |
| **Year** | **2002** | **2003** | **2004** | **2005** | **2006** | **2007** | **2008** | **2009** | **2010** | **2011** | **2012** | **2013** | **2014** | **2015** | **2016** | **2017*** |
| **Active surveillance** |  |  |  |  |  |  |  |  |  |  |  |  |  |  |  |  |
| Healthy slaughter | 19642 | 21140 | 8949 | 8910 | 18564 | 15813 | 10214 | 9905 | 10132 | 11680 | 11454 | 10559 | 0 | 0 | 0 | 0 |
| Fallen stock | 3864 | 4000 | 10137 | 10085 | 17528 | 14990 | 10193 | 10091 | 10094 | 10033 | 10013 | 9625 | 1512 | 1504 | 1520 | 1336 |
| Total | 23506 | 25140 | 19086 | 18995 | 36092 | 30803 | 20407 | 19996 | 20226 | 21713 | 21467 | 20184 | 1512 | 1504 | 1520 | 1336 |
| **Size of sample genotyped** |  |  |  |  |  |  |  |  |  |  |  |  |  |  |  |  |
| Healthy slaughter | 0 | 0 | 0 | 663 | 551 | 1222 | 446 | 483 | 505 | 492 | 562 | 489 | 0 | 0 | 0 | 0 |
| Fallen stock | 0 | 0 | 0 | 433 | 397 | 676 | 446 | 439 | 488 | 411 | 490 | 455 | 751 | 732 | 876 | 927 |
| Total | 0 | 0 | 0 | 1096 | 948 | 1898 | 892 | 922 | 993 | 903 | 1052 | 944 | 751 | 732 | 876 | 927 |
| **Number of classical scrapie cases in active surveillance** | 40 | 51 | 40 | 35 | 38 | 23 | 11 | 3 | 2 | 1 | 0 | 2 | 0 | 0 | 0 | 0 |
| **ARR frequency (%) in genotyped sample** | N/A | N/A | N/A | 37.5 | 50.3 | 47.0 | 54.9 | 61.4 | 63.8 | 64.8 | 67.3 | 69.9 | 73.6 | 77.2 | 77.5 | 76.9 |

Numbers of animals tested in healthy slaughter and fallen stock streams by year; size of the yearly random samples taken for genotyping; number of scrapie cases by year in active surveillance and ARR frequency in the genotyped sample by year.

*until 1 Nov 2017.
